# Influence of Genotype on Endometrial Angiogenesis during Early Pregnancy in Piau and Commercial Line Gilts

**DOI:** 10.3390/ani12050553

**Published:** 2022-02-23

**Authors:** José Carlos Montes-Vergara, Jurandy Mauro Penitente-Filho, Mariana Machado-Neves, Lucas Corrêa Martins Machado, Faider Alberto Castaño-Villadiego, Karine Assis Costa, Eduardo Paulino da Costa, Carolina Filardi de Campos, Camilo José Ramírez-López, Simone Eliza Facioni Guimarães, Paulo Sávio Lopes, José Domingos Guimarães

**Affiliations:** 1 Department of Veterinary, Universidade Federal de Viçosa, Viçosa 36570-900, Brazil; zecave@live.com (J.C.M.-V.); penitentefilho@yahoo.com.br (J.M.P.-F.); lucascmachado7@gmail.com (L.C.M.M.); faider_cas@hotmail.com (F.A.C.-V.); epcosta@ufv.br (E.P.d.C.); camilo2407@gmail.com (C.J.R.-L.); jdguima.ufv@gmail.com (J.D.G.); 2 Department of Biology, Universidade Federal de Viçosa, Viçosa 36570-900, Brazil; machadonevesm@gmail.com; 3 Department of Animal Science, Universidade Federal de Viçosa, Viçosa 36570-900, Brazil; kryneacosta@yahoo.com.br (K.A.C.); carolinafilardi@yahoo.com.br (C.F.d.C.); plopes@ufv.br (P.S.L.)

**Keywords:** genes, pigs, uterus, vascularization

## Abstract

**Simple Summary:**

Angiogenesis occurs physiologically in the maternal–fetal interface, and both maternal and fetal angiogenesis are required for a successful pregnancy. The relative transcript abundance of angiogenesis-related genes in the pig endometrium is controlled by factors that create a microenvironment that promotes successful embryo implantation. Therefore, we investigated the transcript abundance of angiogenesis-related genes in the endometrium of pregnant commercial line and Piau gilts, and their relationship with the phenotypic expression of genes important to the endometrium during early pregnancy (until 30 days). This research aimed to better understand the reproductive development of commercial and local Brazilian breeds, so that it can contribute to the knowledge and practice of pig farming.

**Abstract:**

This study aimed to evaluate the endometrial angiogenesis of pregnant commercial line and Piau gilts during early pregnancy. We used 27 gilts, divided into three groups according to the type of mating: Commercial (*n* = 9), commercial line females mated with commercial line males; Cross-mated (*n* = 9), Piau females mated with commercial line males; and Piau (*n* = 9), Piau females mated with Piau males. Each group was divided into three subgroups based on gestational age at the time of slaughter (7, 15, and 30 days of pregnancy). Immediately after slaughter, endometrial samples were obtained for histological evaluation and for analysis of the relative transcript abundance (RTA) of angiogenesis-related genes (*HIF1α, FGF9, ANG1, TEK, VEGFA, ANGPT1*, and *ANGPT2*). The number of endometrial glands was similar among groups but decreased with gestational age (*p* < 0.05). Piau females showed a higher number of blood vessels (*p* < 0.05) at 7 and 15 days of pregnancy, but no differences were observed among groups at 30 days, suggesting an influence of the male genotype on the pattern of uterine vascularization. There were no differences among groups for RTA of the *FGF9, HIF1α, TEK, VEGFA, ANGPT1*, and *ANGPT2* genes. The *HIF1α*-gene RTA was higher at 7 and 15 days of pregnancy; for *TEK* and *ANGPT1*, the RTA was higher at 15 days of pregnancy; and the RTA of *VEGFA* and *ANGPT2* genes were higher at 30 days of pregnancy. The *ANG1* RTA was similar for pregnancies in the commercial and Piau groups but was higher (*p* < 0.05) at 15 days in the Cross-mated group, suggesting an interaction between genotypes. Overall, the pattern found for the RTA of angiogenesis-related genes was similar among the groups in this study, although some phenotypic differences could be noted, such as the highest number of blood vessels being found during early pregnancy of Piau gilts. The results of the gene RTA when crossed with phenotypic data led to conclusions that are conflicting with those reported in the literature. However, noteworthy is that angiogenesis is a complex process in which the balance between stimulatory and inhibitory factors may be related to time.

## 1. Introduction

In the swine industry, there are genetic groups, such as criolla breeds, that may favorably contribute to breeding programs. Among these groups, the Piau breed has the following characteristics: rusticity, low management requirement, meat and bacon production, and meat marbling [1]. Previous studies carried out have demonstrated that the Piau breed shows a different behavior in terms of reproductive characteristics and probably is high efficient at developing embryos [2,3,4].

Early pregnancy in pigs is characterized by elevated endometrial vascular permeability [5,6], which seems to be associated with the induction of angiogenesis in endometrium [7,8]. The blood flow rate of a pregnant uterus varies during gestation due to differences in the structure and density of the vasculature [9]. The increased embryonic nutrient demand is supplied by increased blood flow as well as the associated increase in angiogenesis and the formation of new blood vessels [10,11].

Angiogenesis occurs physiologically in the maternal–fetal interface and is crucial to the growth and development of a conceptus. Both maternal and fetal angiogenesis are required for a successful pregnancy [12]. The relative transcript abundance (RTA) of angiogenesis-related genes in the pig endometrium is controlled by factors that create a unique microenvironment that promotes successful embryo implantation in this species [8]. Furthermore, endometrial angiogenic support for the placenta is a regulatory means of embryo survival [13]. Several molecules that participate in angiogenic processes are studied here, such as VEGF-A (Vascular Endothelial Growth Factor A), which represents the main vascular development factor [14]. Its transcription is induced by the inducing factor of hypoxia 1 (HIF-1). The angiopoietin system (*ANGPT1* and *ANGPT2*) is involved in cell survival and vascular maturation, and *TEK* (Tyrosine Kinase Endothelial) acts as a receptor for both proteins [15]. The RTA of *FGF-9* (Fibroblastic Growth Factor 9) acts as an important embryonic growth factor [16,17]; moreover, *ANG1* (angiogenin 1) induces angiogenesis through biological processes such as the organization microvascular, stabilization, and survival required for vascular remodeling [18].

Therefore, this study aimed to evaluate the relative transcript abundance of angiogenesis-related genes in the endometrium of pregnant commercial line and Piau gilts, and their relationship with the phenotypic data of the endometrium during early pregnancy (until 30 days). The following hypothesis drives this research: there are differences in the relative transcript abundance and in the uterine phenotypes related to angiogenesis between the Piau breed and the commercial line.

## 2. Materials and Methods

### 2.1. Local

The experiment was conducted at the Pig Breeding Farm, Laboratory of Animal Biotechnology (Labtec, Viçosa, Brazil) and Laboratory of Structural Biology, of the Universidade Federal de Viçosa. The farm is located at an altitude of 660 m and at 20°45′16.3″ S and 42°52′57.02″ W, in Viçosa, MG, Brazil.

### 2.2. Animals and Experimental Design

We used 27 females, eight months of age, among which nine were from a commercial line (Talent^®^, Topigs Norsvin, Oak Bluff, MB, Canada) and 18 were from the local breed Piau.

The females were distributed into three groups according to the type of mating used in the artificial insemination: commercial (*n* = 9), commercial line females inseminated with the semen of commercial line males; cross-mated (*n* = 9), Piau females inseminated with the semen of commercial line males; and Piau (*n* = 9), Piau females inseminated with Piau semen. Each group was subdivided into three subgroups (3 animals each) according to gestational age: 7, 15, and 30 days.

The females were housed in collective bays (25 m^2^) of five animals, and fed rations and supplied water *ad libitum* during the gestational phase.

### 2.3. Artificial Insemination

The females were inseminated 12 and 24 h after the onset of estrus. The females were inseminated with semen from a male of a commercial line (commercial and cross-mated groups) or a Piau male (Piau group) that belonged to the same herd, with proven reproductive efficiency (andrological examination and reproductive history).

The inseminating doses were prepared as a volume of 100 mL (BTS^®^ + semen, Delavan, WI, USA) and a concentration of 3 × 10^9^ sperm/dose and stored at 15 °C for up to 72 h. Artificial insemination was performed by deep intracervical deposition of the semen [19].

### 2.4. Slaughter and Collection of Uterine Samples

The females were slaughtered at a predetermined time (7, 15, and 30 days of pregnancy) after artificial insemination. Uterine samples were collected from the intermediate region of the uterine horn for histological and molecular analyses. The selection of days was a function of the embryonic period, where the transition from the embryonic to fetal phases is completed at around day 35 of pregnancy [20]. The days chosen also represent different milestones in embryonic formation. At 7 days, the embryos are in a blastocyst state floating in the uterine lumen. At 15 days, maternal recognition would have already occurred, the first angiogenic wave would have occurred, and implantation would be close. By day 30, the embryos would be close to transitioning into the fetal stage.

### 2.5. Histological Analysis

Uterine fragments with approximately 2.0 × 0.3 cm were used for histological analysis, preserving the three uterine layers: endometrium, myometrium, and perimetrium. The fragments were fixed by immersion in 4% buffered paraformaldehyde solution [21] for 24 h. Then, they were dehydrated in crescent ethanol series (70, 80, 90, and 100%) and embedded in paraffin. Sections with 4 μM thicknesses were stained with hematoxylin and eosin (HE).

We analyzed the epithelium type lining the endometrium [22]. The endometrial thickness was assessed from images taken from 10 fields randomly chosen per animal at 40 × magnificence; in each field, 10 measurements were performed. In the same fields, the number of endometrial glands in the image was counted. The area of the image was assessed and the number of endometrial glands was adjusted to the number of glands per 10^6^ μm^2^. These analyses were performed using Image Pro Plus software (Version 4.5). The number of blood vessels was counted using a photomicroscope equipped with a digital camera; images from 10 fields randomly chosen per animal were assessed by IScapture Software (Version 3.9, Tucsen, Fuzhou, China) at 100 × magnificence. The area of the image was assessed, and the number of blood vessels was adjusted to the number of vessels per 10^6^ μm^2^. The volume densities of glands and blood vessels were also evaluated by using photomicroscope; images from 30 randomly chosen fields per animal were assessed by ImageJ software (National Institute of Health, Bethesda, MD, USA, https://imagej.nih.gov/ij, accessed on 15 February 2022); a grid with 520 intersections (points) was projected onto each image at 400 × magnificence.

### 2.6. Gene Relative Transcript Abundance Analysis

To carry out the molecular studies, an endometrial sample of approximately 2.0 × 1.0 cm was taken and placed in a 15 mL conical bottom tube with RNA Holder^®^ solution (BioAgency Biotechnology, Toronto, Canada) at room temperature. Subsequently, the samples were incubated at 4 °C for 24 h and stored at −20 °C until processing [23].

The RNA extraction was performed using Trizol (Ambion—Life Technologies, Austin, TX, USA), and synthesis of the first strand was performed using the GoScript™ Reverse Transcription System cDNA kit (Promega^®^, Madison, WI, USA). The cDNA sample concentrations were estimated by spectrophotometry and a single strand of cDNA was stored at −20 °C until use in quantitative PCR in real time (qRT-PCR). Primers (Table 1) for amplification of the target genes (*HIF1α, FGF9, ANG1, TEK, VEGF, ANGPT1*, and *ANGPT2*) and endogenous fragments (*GAPDH*) were designed using the PrimerQuest program provided by Integrated DNA Technologies, Inc. (Coralville, IA, USA) from nucleotide sequences obtained from the GenBank database (http://www.ncbi.nlm.nih.gov, accessed on 1 March 2018).

The gene RTA was measured by qRT-PCR in real time, and for this, the reactions were performed in a thermocycler model ABI Prism 7300 Sequence Detection System (Applied Biosystems, Foster City, CA, USA) using Green^®^ PCR Master Mix SYBR kit (Applied Biosystems, Foster City, CA, USA) according to the manufacturer’s recommendations. The reactions were subjected to a protocol with cycles of the following program: 3 min at 95 °C, 40 cycles at 95 °C for 15 s, and 1 min at 60 °C. All reactions for the same target gene were conducted in duplicates.

The data obtained from the qRT-PCR reaction were generated as Ct values and were calculated from ΔCt (target Ct—endogenous reference Ct) to minimize the possible variations in the amount of starting mRNA and the efficiency during reverse transcription. The relative transcript abundance of the target gene (2^−ΔCt^) was calculated [24,25].

### 2.7. Statistical Analysis

The experiment was carried out in a completely randomized design, according to the following mathematical model:

(1) $$Y_{ijk}=\mu +G_{i}+D_{j}+{\left(GD\right)}_{ij}+e_{ijk}$$

where $Y_{ijk}$ is the response, μ is a constant, $G_{i}$ is the effect of the group, $D_{j}$ is the effect of the day of pregnancy, ${\left(GD\right)}_{ij}$ is the interaction, and $e_{ijk}$ is the error.

The data for the volume densities of glands and blood vessels were arcsine transformed ($y^{\prime }=arcsin\sqrt{y}$) and analyzed by ANOVA (GLM Procedure) [26], and a comparison among means (LS-means) was performed using the Tukey–Kramer test. The relationship among variables was verified by Pearson’s simple correlation (CORR procedure). The significance level adopted was α = 0.05.

## 3. Results

**Figure 1:**
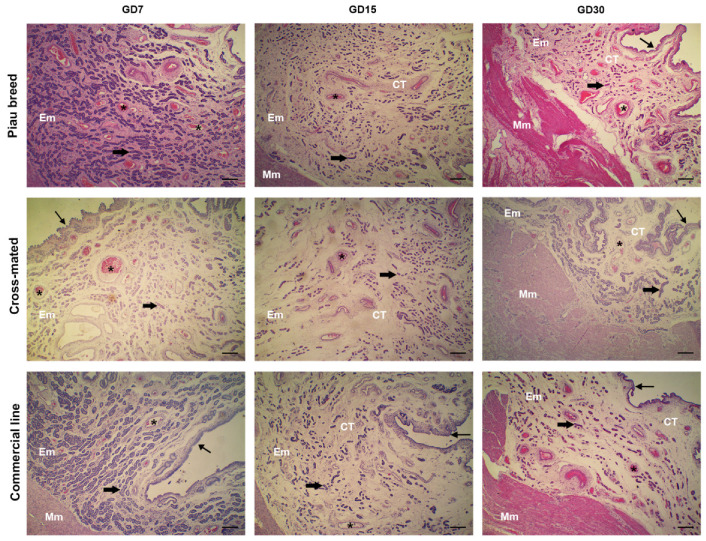
Histological sections of the uterus from the Piau breed, cross-mated (Piau breed x commercial line), and commercial line females at 7, 15, and 30 days of gestation (GD). The endometrium (Em) was composed of an epithelium (thin arrow) supported by a connective tissue (CT) with blood vessels (*) and endometrial glands (thick arrows). Note the presence of myometrium (Mm) composed of smooth muscle cells. Hematoxylin and eosin. Scale bar: 50 µM.

The histological analysis showed a columnar pseudostratified epithelium lining the endometrium, with focal areas from a single layer of the columnar epithelium. Moreover, we observed a lamina propria and submucosa composed of a thin subepithelial vascular layer of loose connective tissue, and a glandular layer composed of tubular endometrial glands surrounded by connective tissue near the myometrium (Figure 1).

**Figure 2:**
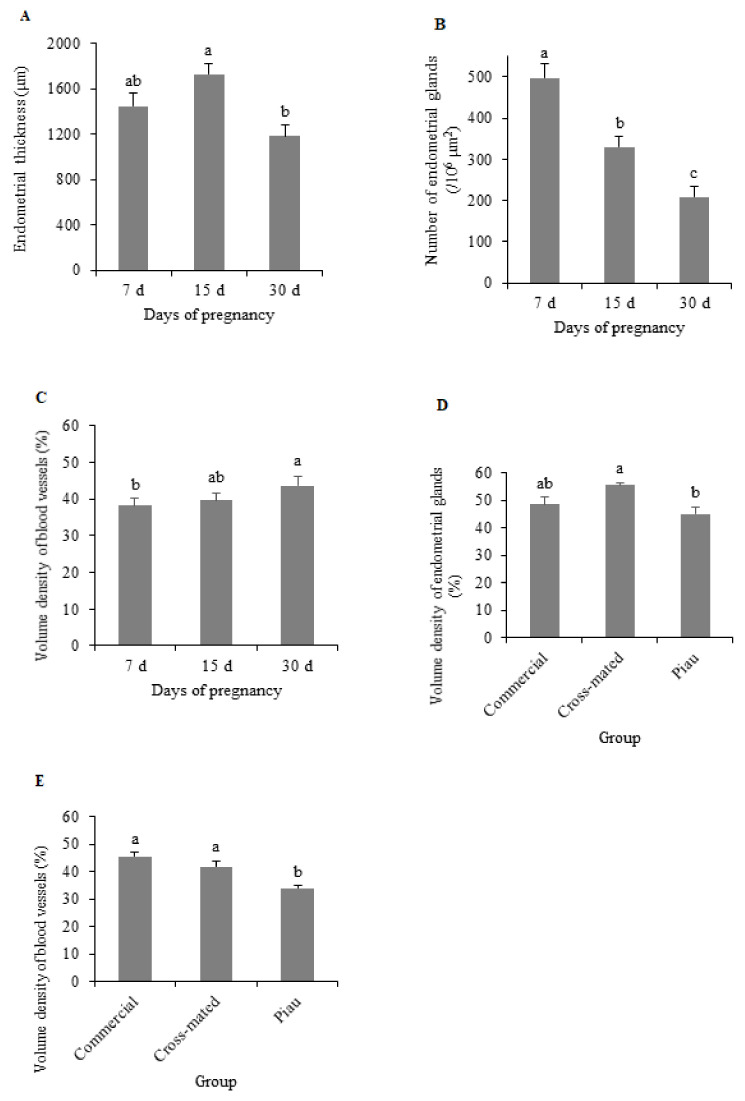
Endometrial thickness (**A**), number of endometrial glands (**B**), and volume density of blood vessel (**C**) according to days of pregnancy, and volume density of endometrial glands (**D**) and blood vessels (**E**) according to the group with commercial gilts and Piau breed gilts mated with commercial males (cross-mated) and Piau breed males (Piau). The results are presented as days of pregnancy (**A**–**C**) or group (**D**,**E**) since there is no interaction with these variables. Different letters indicate differences at *p <* 0.05. Bars, SE.

Endometrial thickness, number, and volume density of the endometrial glands and the blood vessels showed no interaction between group and day of pregnancy (*p >* 0.05); nevertheless, the number of endometrial glands decreased during pregnancy (*p <* 0.05), although a clear pattern was not observed for endometrial thickness, and the volume density of the blood vessels increased from the 7th to 30th day of pregnancy. The percentages of endometrial glands and blood vessels were smaller in the Piau group (*p* < 0.05; Figure 2).

There was an interaction between groups and days of pregnancy (*p <* 0.05) for the number of blood vessels. Overall, both groups of Piau females showed a higher number of blood vessels at 7 and 15 days of pregnancy, but no differences were observed among the groups at 30 days (Table 2).

**Figure 3:**
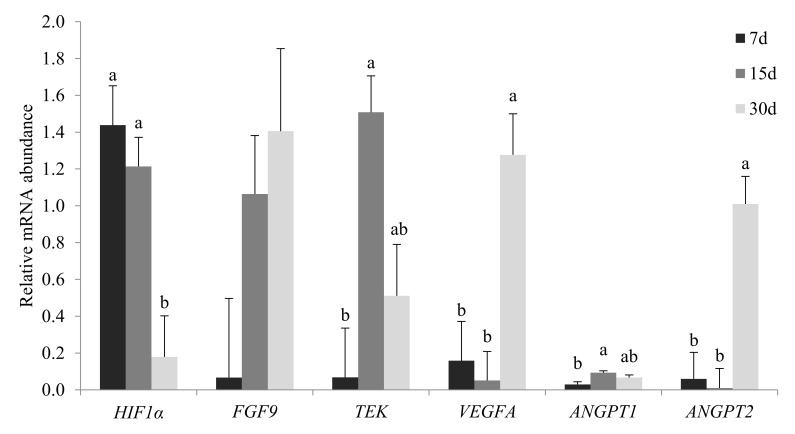
RTA of the *HIF1α, FGF9, TEK, VEGFA, ANGPT1,* and *ANGPT2* genes according to gestational age in commercial and Piau breed gilts. Different letters indicate differences at *p <* 0.05.

There was no effect of group, days of pregnancy, or interaction for the *FGF9* gene RTA (*p >* 0.05). The day of pregnancy influenced the RTA of the *HIF1α, TEK, VEGFA, ANGPT1*, and *ANGPT2* genes (*p* < 0.05; Figure 3).

**Figure 4:**
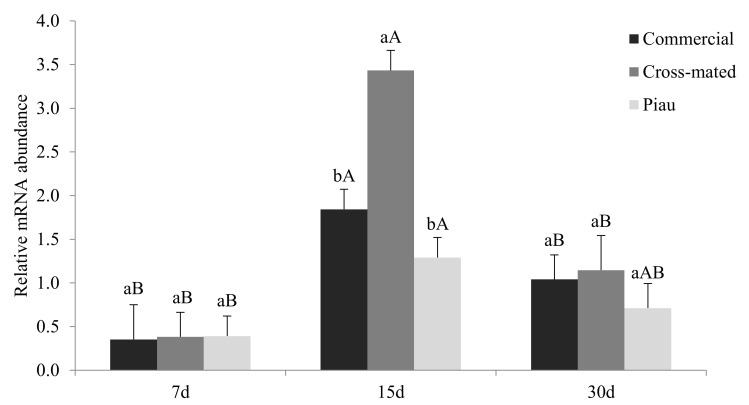
RTA of the *ANG1* gene according to gestational age in commercial gilts and Piau breed gilts mated with commercial males (cross-mated) and Piau breed males (Piau). Within the days of pregnancy, different lowercase letters indicate differences among breeds (*p <* 0.05). Within groups, different uppercase letters indicate differences among days of pregnancy (*p <* 0.05). Bars, SE.

There was an interaction (*p <* 0.05) between groups and days of pregnancy for RTA of the *ANG1* gene (Figure 4). Females of the cross-mated group presented higher *ANG1* RTA at 15 days of pregnancy, but there was no difference among groups at 7 and 30 days.

The number of blood vessels was negatively correlated with *ANGPT1* RTA (*r* = −0.582). The number of endometrial glands was negatively correlated with *VEGFA* RTA (*r* = −0.606) and *ANGPT2* (*r* = −0.641) genes and positively correlated to *HIF1α* RTA (*r* = 0.510). The volume density of blood vessels was positively correlated with *FGF9* RTA (*r* = 0.520). The endometrial thickness was negatively correlated with the RTA of *VEGFA* (*r* = −0.597) and *ANGPT2* (*r* = −0.643) genes.

## 4. Discussion

This study was developed to evaluate the abundance of angiogenesis-related gene transcripts in the pregnant endometrium of pigs from two different genetic groups, commercial line and Piau gilts, and their relationship with phenotypic data of the endometrium during early pregnancy (until 30 days). During the embryonic stage of pregnancy, communication between the endometrium and embryos is crucial in their development [27]. Therefore, endometrial glands become essential at this stage to meet the nutritional needs of embryos [28] at the beginning of pregnancy. In this study, the number of endometrial glands decreased during pregnancy and the endometrial thickness decreased from day 15 to day 30 in both commercial and Piau breed, suggesting that both genotypes present similar patterns of endometrial development during early pregnancy.

However, the vascularization pattern was markedly different among groups. Piau gilts mated to Piau males presented a higher number of blood vessels than commercial gilts and Piau gilts mated to commercial males (cross-mated group) at 7 and 15 days of pregnancy; however, the volume density of blood vessels was lower in the Piau group. These findings may suggest that the embryo genotype may influence the vascularization pattern in early pregnancy in Piau gilts.

Between 15 and 20 days of pregnancy, there is a gradual transition in the embryo nutrition, from hystotrophic to hemotrophic, which is dependent on the interactions between the endometrium and the placenta [29]. With the formation of the placenta, there is reduction in the number of endometrial glands as the pregnancy advances; angiogenic support in early pregnancy is involved in creating a favorable uterine microenvironment for implementation and other events in the embryonic stage [8]. In a previous study [4], we reported that the embryo survival rate was higher in the Piau breed than in commercial gilts. In this study, the percentage of blood vessels was lower but the number of blood vessels was higher in the Piau group, suggesting a different pattern of vascularization in early pregnancy that may contribute to a more favorable uterine environment for early embryo development.

In the present study, the *HIF1α* gene showed higher RTA in early pregnancy and decreased as the pregnancy advanced in all groups evaluated. This gene plays a key role in regulating hypoxic adaptation by activating the transcription of genes involved in angiogenesis, erythropoiesis, and glycolysis [30,31] and regulates the peri-implantation angiogenesis in gilts with high RTA at 20 days of pregnancy [32].

In this study *HIF1α* RTA was positively correlated with number of endometrial glands. The *HIF1α* gene plays a role in the cell differentiation of mouse mammary gland [33], but its role in regulating endometrial glands development still needs clarification.

The *HIF1α* gene is also a transcription inducer of *VEGFA* [34,35], which is considered the primary vascular development factor [14]. However, in this study, the RTA of the *VEGFA* gene had opposite trend compared with *HIF1α* gene during pregnancy, with a lower RTA at 7 and 15 days and a higher at 30 days of pregnancy. This suggests a different pathway for stimulation of the *VEGFA* gene in swine endometria. Several mechanisms may be involved in the regulation of *VEGF* RTA; many cytokines, growth factors, and hormones, such as transforming growth factor (TGF)-α, basic fibroblast growth factor (bFGF), intelerukin-6 (IL-6), and follicle stimulating hormone (FSH) could also induce *VEGF* RTA in various tissues [36,37]; nevertheless, the signaling pathway for *VEGFA* expression in swine endometrial during early pregnancy still remains unclear.

The negative correlation between *VEGFA* RTA and the number of endometrial glands was an unexpected result; it has been reported that VEGF inactivation leads to failure in mice mammary gland development and function [38]. Nevertheless, this finding may not suggest that *VEGFA* inhibits endometrial glands development but may lead to the conclusion that it is not involved in swine endometrial gland development in early pregnancy. This result may be explained by the fact that the number of endometrial glands decreases as pregnancy advances [8], which coincides with the lower levels of *VEGFA* RTA in early pregnancy (7–15 days), which increases at 30 days. The transition in embryo nutrition, from hystotrophic to hemotrophic, depends on interactions between the endometrium and the placenta [29]; the *VEGF* is expressed in placental tissues and fetal membranes; and this expression increases with advancing gestation [39].

The angiopoietin system is involved in endothelial cell survival and vascular maturation and involves interactions of *ANGPT1* and *ANGPT2* with *TEK*, the receptor for both proteins [40]. The *ANGPT1* has been reported to be an inducer of vascularization by increasing the number of vessels [41] and/or promoting an enlargement of the existing vessels without increasing the number of vessels [42]. In this study, *ANGPT1* RTA was slightly higher at day 15 of pregnancy. Additionally, the negative correlation between *ANGPT1* RTA and number of endometrial blood vessels was an unexpected result; this may suggest a non-essential role of *ANGPT1* gene in endometrial angiogenesis in the early pregnancy of gilts. However, further investigations are still needed to clarify these findings. Low-level and paracrine *ANGPT1* RTA controls vascular quiescence and maintains the resting and anti-adhesive state of the vascular endothelium promoting blood vessel stabilization [43]; this could explain the steady number of endometrial blood vessels during pregnancy in commercial and cross-mated groups. However, the role of *ANGPT1* in the Piau group, in which the number of blood vessels decreased during pregnancy, still remains elusive.

*ANGPT2* was reported as a competitive inhibitor for *ANGPT1* due to its binding to *TEK* without receptor activation [44]. It destabilizes mature blood vessels, leading to either angiogenesis or vascular regression, depending on the presence or absence of *VEGFA*, respectively [45,46]. In this study, *ANGPT2* RTA was lower at 7 and 15 days and higher at 30 days of pregnancy. In commercial and cross-mated groups, the number of blood vessels remained the same during pregnancy regardless of the *ANGPT2* RTA; however, for the Piau group, the decrease in the number of blood vessels was accompanied by an increase in *ANGPT2* RTA. This was contradictory to the increase in *VEGFA* RTA at 30 days, which would lead to the stimulation of angiogenesis, although the *TEK* RTA was lower at 30 days. Therefore, the results of this study indicate the need for further investigations regarding to the role of the *ANGPT1/2/TEK* system on endometrial angiogenesis control, especially in Piau gilts.

Angiogenin (*ANG1*) is a ribonuclease (RNase) superfamily member, and it is contained in a vasculature that rarely undergoes proliferation, but in some physiological conditions, its levels increase in blood, promoting neovascularization [47]. In this study, *ANG1* RTA was markedly higher at 15 days of pregnancy in the cross-mated group. This period coincides with the transition from hytologic to hemotrophic embryo nutrition [29]; with the beginning of the wave of angiogenesis [5]; and almost with the stage of embryonic development, in which it gives rise to a tubular structure about 15 cm long [48,49] and the elongation pattern seems to be different among Piau, commercial, and cross-mated embryos [4]. Since *ANG1* is secreted under stress conditions, such as hypoxia [50], our findings suggest that, in Piau gilts cross-mated with commercial males, there is a type of adaptation of the endometrium to accommodate embryo survival. In fact, we demonstrated that embryo survival rates at 15 days of pregnancy are similar in Piau gilts mated with Piau males or commercial males [4].

## 5. Conclusions

Overall, the patterns of the relative transcript abundance of angiogenesis-related genes in this study were similar among pregnant commercial and Piau breed gilts on different days during early pregnancy, although some phenotypic differences could be noted, such as the highest number of blood vessels being found in early pregnancies of Piau gilts; this difference was probably due to the influence of the male genotype since cross-mated Piau gilts showed the same pattern as commercial gilts. An interaction between genotypes could also be noted in *ANG1* RTA and was higher in cross-mated Piau gilts. The results of the gene expression when crossed with phenotypic data led to conclusions that are in conflict with that in the literature. However, it is noteworthy that angiogenesis is a delicate and complex process in which the balance between stimulatory and inhibitory factors may be related to time. Therefore, the findings of the present study reinforce the need for further investigations in endometrial angiogenesis of pigs.

## Figures and Tables

**Table 1:** Primers used for amplification in real-time PCR reactions.

Gene	Protein	Access Number	Primer Sequence
*HIF1α*	Hypoxia inducer factor-1α	NM_001123124.1	F: GCCAGATCTCGACGAAGTAAAG R: AGCTGATGGTAAGCCTCATAAC
*FGF9*	Fibroblast growth factor-9	NM_213801.1	F: CAGTCACGGACTTGGATCATT R: TTCCTGGTTCCCTGGATAGT
*ANG1*	Angiogenin-1	NM_001044573.2	F: GAAGACAGGTACACACACTTCC R: CAGGCCTCGTTGCTTCATTA
*TEK*	Tyrosine endothelial kinase	XM_001926034.5	F: CGGCACGAAGTACCTGATATT R: GGTGAAGAGGTTTCCTCCTATG
*VEGFA*	Vascular endothelial growth factor A	NM_214084.1	F: GCACATAGGAGAGATGAGCTTC R: CAAGGCCCACAGGGATTT
*ANGPT1*	Angiopoietin-1	NM_213959.1	F: ACAGAGCCACCACCAATAAC R: GTGCAAAGGTTGACGAGATTATG
*ANGPT2*	Angiopoietin-2	NM_213808.1	F: CTGAGCTGTGATCTCGTCTTG R: CTGAACCTGATACTGCCTCTTC
*GAPDH*	Glyceraldehyde 3-phosphate dehydrogenase	NM_001206359.1	F: CAAAGTGGACATTGTCGCCATCA R: AGCTTCCCATTCTCAGCCTTGACT

**Table 2:** Number of blood vessels (per 10^6^ μm^2^) according to the days of pregnancy in commercial gilts and Piau breed gilts mated with commercial males (cross-mated) and Piau breed males (Piau) (Mean ± SE).

Days of Pregnancy	Commercial	Cross-Mated	Piau
7	78.5 ± 11.8 ^bA^	72.8 ± 6.8 ^bA^	136.6 ± 6.8 ^aA^
15	51.5 ± 6.8 ^bA^	69.3 ± 6.8 ^bA^	112.2 ± 6.8 ^aA^
30	66.1 ± 6.8 ^aA^	73.4 ± 6.8 ^aA^	77.2 ± 6.8 ^aB^

Different lowercase letters within a row and uppercase letters within a column differ at *p <* 0.05.

## Data Availability

The data presented in this study are available from the corresponding author upon request.

## References

[1] Sollero B.P., Paiva S.R., Faria D.A., Guimarães S.E.F., Castro S.T.R., Egito A.A., Albuquerque M.S.M., Piovezan U., Bertani G.R., Mariante A.D.S. (2009). Genetic diversity of Brazilian pig breeds evidenced by microsatellite markers. Livest. Sci.

[2] Silva P.V., Guimarães S.E.F., Guimarães J.D., Neto J.B., Lopes P.S., Nascimento C.S.D., De Campos C.F., A Weller M.M.C., E Botelho M., Faria V.R. (2011). Gene expression in swine granulosa cells and ovarian tissue during the estrous cycle. Genet. Mol. Res..

[3] Silva P.V., Guimarães S.E.F., Guimarães J.D., Nascimento C.S., Lopes P.S., Siqueira J.B., Amorim L.S., E Silva F.F., Foxcroft G.R. (2014). Follicular dynamics and gene expression in granulosa cells, corpora lutea and oocytes from gilts of breeds with low and high ovulation rates. Reprod. Fertil. Dev..

[4] Montes J.C., Penitente-Filho J.M., Guimarães S.E.F., Lopes P.S., Camilo B.S., Shiomi H.H., Lima D.A., Pinho R.O., Pereira J.V.T.D.N., Okano D.S. (2018). Aspects of sexual precocity and morphometry of uterus, placenta and embryos/fetuses in Piau breed and Commercial line gilts. Theriogenology.

[5] Keys J.L., King G.J., Kennedy T.G. (1986). Increased uterine vascular permeability at the time of embryonic attachment in the pig. Biol. Reprod..

[6] Keys J.L., King G.J. (1988). Morphological evidence for increased uterine vascular permeability at the time of embryonic attachment in the pig. Biol. Reprod..

[7] Dvorak H.F., Brown L.F., Detmar M., Dvorak A.M. (1995). Vascular permeability factor/vascular endothelial growth factor, microvascular hyperpermeability, and angiogenesis. Am. J. Pathol..

[8] Kaczmarek M.M., Kiewisz J., Schams D., Ziecik A.J. (2009). Expression of VEGF-receptor system in conceptus during peri-implantation period and endometrial and luteal expression of soluble VEGFR-1 in the pig. Theriogenology.

[9] Ford S.P., Vonnahme K.A., Wilson M.E. (2002). Uterine capacity in the pig reflects a combination of uterine environment and conceptus genotype effects. J. Anim Sci.

[10] Reynolds L.P., Borowicz P.P., Caton J.S., Vonnahme K.A., Luther J.S., Buchanan D.S., Hafez S.A., Grazul-Bilska A.T., Redmer D.A. (2010). Uteroplacental vascular development and placental function: An update. Int. J. Dev. Biol..

[11] Reynolds L.P., Borowicz P.P., Caton J.S., Vonnahme K.A., Luther J.S., Hammer C.J., Maddock Carlin K.R., Grazulbilska A.T., Redmer D.A. (2010). Development programming: The concept, large animal models, and the key role of uteroplacental vascular development. J. Anim. Sci..

[12] Tayade C., Fang Y., Croy B.A. (2007). A review of gene expression in porcine endometrial lymphocytes, endothelium and trophoblast during pregnancy success and failure. J. Reprod. Dev..

[13] Wessels J., Linton N.F., Croy B.A., Tayade C. (2007). A review of molecular contrast between arresting and viable porcine attachment sites. Am. J. Reprod. Immunol..

[14] Tammela T., Enholm B., Alitalo K., Paavonen K. (2005). The biology of vascular endothelial growth factors. Cardiovasc. Res..

[15] Oliver G., Novak S., Patterson J.L., Pasternak J.A., Paradis F., Norrby M., Oxtoby K., Dyck M.K., Dixon W.T., Foxcroft G.R. (2011). Restricted feed intake in lactating primiparous sows: II. Effects on subsequente litter sex ratio and embryonic gene expression. Reprod. Fertil. Dev..

[16] Østrup E., Bauersachs S., Blum H., Wolf E., Hyttel P. (2010). Differential endometrial gene expression in pregnant and nonpregnant sows. Biol. Reprod..

[17] Samborski A., Graf A., Krebs S., Kessler B., Bauersachs S. (2013). Deep Sequencing of the Porcine Endometrial Transcriptome on Day 14 of Pregnancy. Biol. Reprod..

[18] Bazer F.W., Wu G., Spencer T.E., Johnson G.A., Burghardt R.C., Bayless K. (2010). Novel pathways for implantation and establishment and maintenance of pregnancy in mammals. Mol. Hum. Reprod..

[19] Serret C.G., Alvarenga M.V.F.D., Coria A.L.P., Dias C.P., Corcini C.D., Corrêa M.N., Deschamps J.C., Bianchi I., Lucia T. (2005). Intrauterine artificial insemination of swine with different sperm concentrations, parities, and methods for prediction of ovulation. Anim. Reprod..

[20] Panzardi A., Megalli P.G., Bernardi M., Wentz I., Bortolozzo F.P. (2007). Eventos cronológicos da gestação: Da deposição dos espermatozoides no trato reprodutivo feminino ao desenvolvimento dos fetos. Suinocultura em Ação: A Fêmea Suína Gestante.

[21] Vonnahme K.A., Ford S.P. (2003). Placental vascular endothelial growth factor receptor system mRNA expression in pigs selected for placental efficiency. J. Physiol..

[22] Monteiro C.M.R., Carvalho R.G. (2006). Caracterização histológica do útero, tubas uterinas e ovários de fêmeas recém-nascidas, pré-púberes e púberes de suínos mestiços (*Sus scrofa domestica*–L.1758). ARS Veterinaria.

[23] Skowronski M.T., Kwon T.H., Nielsen S. (2009). Immunolocalization of aquaporin 1, 5, and 9 in the female pig reproductive system. J. Histochem. Cytochem..

[24] Pfaffl M.W. (2001). A new mathematical model for relative quantification in real-time RT-PCR. Nucleic Acids Res..

[25] Livak K.J., Schmittgen T.D. (2001). Analysis of Relative Gene Expression Data Using Real-Time Quantitative PCR and the −2^ΔΔCT^ Method. Methods.

[26] SAS Institute Inc (2002). SAS/STAT^®^ 9.0 User’s Guide.

[27] Spencer T.E., Hayashi K., Hu J., Carpenter K.D. (2005). Comparative development biology of mammalian uterus. Curr. Top. Dev. Biol..

[28] Spencer T.E., Bazer F.W. (2004). Uterine and placental factors regulating conceptus growth in domestic animals. J. Anim. Sci..

[29] Strobant H.W., Taverne N., Lagenfeld K., Barends P.M. (1986). The ultrastructure of the uterine epithelium of the pig during the estrous cycle and early pregnancy. Cell Tissue Res..

[30] Seagroves T.N., Ryan H.E., Lu H., Wouters B.G., Knapp M., Thibault P., Laderoute K., Johnson R.S. (2001). Transcription Factor HIF-1 is a necessary mediator of the Pasteur Effect in mammalian cells. Mol. Cell Biol..

[31] Masson N., Ratcliffe P.J. (2003). HIF prolyl and asparaginyl hydroxylases in the biological response to intracellular O_2_ levels. J. Cell Sci..

[32] Tayade C., Fang Y., Hilchie D., Croy B.A. (2007). Lymphocyte contributions to altered endometrial angiogenesis during early and midgestation fetal loss. J. Leukoc. Biol..

[33] Seagroves T.N., Hadsell D., McManaman J., Palmer C., Liao D., McNulty W., Welm B., Wagner K.U., Neville M., Johnson R.S. (2003). HIF1alpha is a critical regulator of secretory differentiation and activation, but not vascular expansion, in the mouse mammary gland. Development.

[34] Valdés G., Corthorn J. (2011). Review: The angiogenic and vasodilatory utero-placental network. Placenta.

[35] Shan B., Gerez J., Haedo M., Fuertes M., Theodoropoulou M., Buchfelder M., Losa M., Stalla G.K., Arzt E., Renner U. (2012). RSUME is implicated in HIF-1-induced VEGF-A production in pituitary tumour cells. Endocr. Relat. Cancer.

[36] Song K.H., Song J., Jeong G.B., Kim J.M., Jung S.H., Song J. (2001). Vascular endothelial growth factor- its relation to neovascularization and their significance as prognostic factors in renal cell carcinoma. Yonsei Med. J..

[37] Shin S.Y., Lee H.J., Ko D.S., Lee H.C., Park W.I. (2005). The regulators of VEGF expression in mouse ovaries. Yonsei Med. J..

[38] Rossiter H., Barresi C., Ghannadan M., Gruber F., Mildner M., Födinger D., Tschachler E. (2007). Inactivation of VEGF in mammary gland epithelium severely compromises mammary gland development and function. FASEB J..

[39] Cheung C.Y. (1997). Vascular endothelial growth factor: Possible role in fetal development and placental function. J. Soc. Gynecol. Investig..

[40] Milkiewicz M., Ispanovic E., Doyle J.L., Haas T.L. (2006). Regulators of angiogenesis and strategies for their respective manipulation. Int. J. Biochem. Cell Biol..

[41] Suri C., McClain J., Thurston G., McDonald D.M., Zhou H., Oldmixon E.H., Sato T.N., Yancopoulos G.D. (1998). Increased vascularization in mice overexpressing angiopoietin-1. Science.

[42] Thurston G. (2002). Complementary actions of VEGF and Angiopoietin-1 on blood vessel growth and leakage. J. Anat..

[43] Augustin H.G., Koh G.Y., Thurston G., Alitalo K. (2009). Control of vascular morphogenesis and homeostasis through the angiopoietin–Tie system. Nat. Rev. Mol. Cell Biol..

[44] Maisonpierre P.C., Suri C., Jones P.F., Bartunkova S., Wiegand S.J., Radziejewski C., Compton D., McClain J., Aldrich T.H., Papadopoulos N. (1997). Angiopoietin-2, a natural antagonist for Tie2 that disrupts in vivo angiogenesis. Science.

[45] Lobov I.B., Brooks P.C., Lang R.A. (2002). Angiopoietin-2 displays VEGF-dependent modulation of capillary structure and endothelial cell survival in vivo. Proc. Natl. Acad. Sci. USA.

[46] De Spiegelaere W., Cornillie P., Simoens P., Van den Broeck W. (2011). Immunohistochemical detection of the angiopoietins during porcine metanephric kidney development. Acta Histochem..

[47] Tello-Montoliu A., Patel J.V., Lip G.Y.H. (2006). Angiogenin: A review of the pathophysiology and potential clinical applications. J. Thromb. Haemost..

[48] Perry J.S., Rowlands I.W. (1962). Early pregnancy in the pig. J. Reprod. Fertil..

[49] Geisert R.D., Renegar R.H., Thatcher W.W., Roberts R.M., Bazer F.W. (1982). Establishment of pregnancy in pig: II. Cellular remodeling of the porcine blastocyst during elongation on day 12 of pregnancy. Biol. Reprod..

[50] Rajashekhar G., Loganath A., Roy A.C., Chong S.S., Wong Y.C. (2005). Hypoxia up-regulated angiogenin and down-regulated vascular cell adhesion molecule-1 expression and secretion in human placentaltrophoblasts. J. Soc. Gynecol. Investig..

